# Calcium Supplements and Risk of Cardiovascular Disease: A Meta-Analysis of Clinical Trials

**DOI:** 10.3390/nu13020368

**Published:** 2021-01-26

**Authors:** Seung-Kwon Myung, Hong-Bae Kim, Yong-Jae Lee, Yoon-Jung Choi, Seung-Won Oh

**Affiliations:** 1 Department of Cancer Biomedical Science, National Cancer Center Graduate School of Cancer Science and Policy, Goyang 10408, Korea; 2 Cancer Epidemiology Branch, Division of Cancer Epidemiology and Prevention, Research Institute, National Cancer Center, Goyang 10408, Korea; 3 Department of Family Medicine and Center for Cancer Prevention and Detection, Hospital, National Cancer Center, Goyang 10408, Korea; 4 Department of Family Medicine, MyongJi Hospital, Hanyang University College of Medicine, Goyang 10475, Korea; hongbai96@mjh.or.kr; 5 Department of Family Medicine, College of Medicine, Yonsei University, Seoul 03722, Korea; UKYJHOME@yuhs.ac; 6 Department of Preventive Medicine, Seoul National University College of Medicine, Seoul 03080, Korea; hierica8@snu.ac.kr; 7 Department of Family Medicine, Healthcare System Gangnam Center, Seoul National University Hospital, Seoul 06236, Korea; sw.oh@snu.ac.kr

**Keywords:** calcium supplements, cardiovascular disease, randomized controlled trials, meta-analysis

## Abstract

Background: Recent systematic reviews and meta-analyses of randomized, double-blind, placebo-controlled trials (double-blind, placebo-controlled RCTs) have reported controversial findings regarding the associations between calcium supplements on the risk of cardiovascular disease (CVD). This meta-analysis aimed to investigate the association between them. Methods: We searched PubMed, EMBASE, the Cochrane Library, and the bibliographies of relevant articles for double-blind, placebo-controlled RCTs in November, 2020. Relative risks (RRs) with 95% confidence intervals (CIs) for the risk of cardiovascular disease were calculated using a random effects model. The main outcomes were CVD, coronary heart disease (CHD), and cerebrovascular disease. Results: A total of 13 double-blind, placebo-controlled RCTs (*n* = 28,935 participants in an intervention group and 14,243 in a control group)) were included in the final analysis. Calcium supplements significantly increased the risk of CVD (RR 1.15, 95% CI 1.06–1.25], I2 = 0.0%, *n* = 14) and CHD (RR 1.16, 95% CI 1.05–1.28], I2 = 0.0%, *n* = 9) in double-blind, placebo-controlled RCTs, specifically in healthy postmenopausal women. In the subgroup meta-analysis, dietary calcium intake of 700–1000 mg per day or supplementary calcium intake of 1000 mg per day significantly increased the risk of CVD and CHD. Conclusions: The current meta-analysis found that calcium supplements increased a risk of CVD by about 15% in healthy postmenopausal women.

## 1. Introduction

Current guidelines for the prevention and treatment of osteoporosis recommend adequate intakes of dietary calcium ranging 700–1200 mg/day for adults aged 50 and older from health and academic organizations such as the National Osteoporosis Foundation in 2014, American Association of Clinical Endocrinologists and American College of Endocrinology in 2016, and National Osteoporosis Guideline Group in 2017 [1,2,3]. If dietary intakes are insufficient, calcium supplements are recommended. However, Bolland et al. raised concerns that calcium supplements were associated with an increased risk of myocardial infarction by about 30% in their meta-analysis of randomized, double-blind, placebo-controlled trials (double-blind, placebo-controlled RCTs) in 2010 [4] and in their updated meta-analysis published in 2011 [5]. Lewis et al. reported that there was no significant association between calcium supplementation and the risk of coronary heart disease (CHD) in the meta-analysis of randomized controlled trials [6], claiming that none of the trials included in Bolland et al.’s meta-analysis had cardiovascular disease (CVD) as its primary endpoint, and over 65% of all the heart attacks were self-reported [7]. In response to Lewis et al.’s criticism, Bolland et al. asserted that the results did not change when they adjusted for potential confounders in secondary analyses, considerable amounts of data on heart attacks were obtained from death certificates and medical records [8], and the results of Lewis et al.’s meta-analysis were similar to those from their previous meta-analysis, when an open-label study was excluded [9].

In the meantime, based on the existing peer-reviewed scientific literature, including Chung et al.’s updated systematic review and meta-analysis that there was no significant difference between calcium supplementation with or without vitamin D supplementation [10], the National Osteoporosis Foundation and the American Society for Preventive Cardiology announced a clinical guideline that there is moderate-quality evidence that calcium with or without vitamin D intake from food or supplements has no association with CVD in generally healthy adults [11].

In sum, recent systematic reviews and meta-analyses of double-blind, placebo-controlled RCTs have reported controversial findings regarding the associations between calcium supplements on the risk of CVD. Thus, we investigated the associations between the use of calcium supplements and the risk of CVD by conducting a comprehensive meta-analysis of double-blind, placebo-controlled RCTs with various subgroup analyses according to important factors that can affect the results and also compared the differences in main findings and conclusions between the previous systematic reviews and meta-analyses and the current study.

## 2. Materials and Methods

### 2.1. Data Sources and Search

We searched PubMed, EMBASE, and the Cochrane Library using common keywords related to calcium supplements and the risk of CVD from inception to November, 2020 without language restrictions. The keywords were as follows: “calcium supplements,” “calcium supplementation,” and “calcium intake,” for exposure factors; “cardiovascular disease,” “coronary heart disease,” “ischemic heart disease,” “myocardial infarction,” “angina,” “heart failure,” “cerebrovascular disease,” and “stroke” for outcome factors; “randomized controlled study” for study design. We combined the above search terms with AND and OR. Calcium supplementation was defined as the use of elemental calcium supplements of at least 500 mg/d, which is a conventionally used dosage, as carbonate, citrate, or gluconate. We reviewed the bibliographies of relevant articles to find out additional publications from the previous review articles and reference lists. We did not conduct a grey literature search.

### 2.2. Study Selection and Eligibility

Using the PICO (patient, problem or population; intervention; comparison, control or comparator; and outcome) criteria for eligibility criteria, we included double-blind, placebo-controlled RCTs that investigated the associations between the use of calcium supplements and the risk of cardiovascular events or mortality in adults. In this meta-analysis, we excluded open-label trials such as randomized controlled trials that did not use placebos as a control group. In the case of duplicated or shared data from the same population, we included the more comprehensive study or the most recently published one with a longer follow-up in the analysis.

Two of the authors (H.-B.K. and Y.-J.L.) independently assessed the eligibility of all studies based on the pre-determined selection criteria. Disagreements between evaluators were resolved by discussion or in consultation with a third author (S.-K.M.).

### 2.3. Assessment of the Risk of Bias

We estimated the risk of bias based on the Cochrane Risk of Bias Tool [12] by two authors (Y.-J.C. and S.-K.M.), and those with low risk of bias (≥5 items) were considered as an overall low risk of bias study in this analysis.

### 2.4. Main and Subgroup Analysis

For the main analysis, we investigated the associations between the use of calcium supplements and the risk of CVD, CHD, and cerebrovascular disease. CVDs are a group of disorders of the heart and blood vessels and include mainly CHD (disease of the blood vessels supplying the heart muscle, e.g., angina, myocardial infarction, and heart failure) and cerebrovascular disease (disease of the blood vessels supplying the brain, e.g., ischemic and hemorrhagic stroke). Additionally, in order to estimate the increase in absolute risk associated with calcium supplementation, we calculated the absolute risk difference by using the formula, ‘absolute risk difference = baseline risk (RR-1)’: Baseline risk is the number of CVD or CAD events divided by the number of study participants in the placebo or control group. Subgroup meta-analyses were performed according to various factors for each disease outcome (CVD, CHD, and cerebrovascular disease) as follows: incidence or mortality, type of study population (healthy postmenopausal women and subjects with underlying disease), type of disease outcome, gender (men, women, and both), mean age of subjects (<55 years and ≥55 years), region (North America, Europe, and Asia), follow-up period (≤10 years and >10 years), dosage of calcium supplements, and adjustment for dietary calcium intake (no and yes) for prospective cohort studies; incidence or mortality, gender (men, women, and both), mean age of subjects (<65 years and ≥65 years), region (Oceania, North America, and Europe), type of calcium preparation (carbonate, citrate, mixed, and not specified), duration of calcium supplementation (<5 years and ≥5 years), dosage of calcium supplements (500–600 mg/d, 1000 mg/d, ≤1000 mg/d, and >1000 mg/d), concurrent use of vitamin D (calcium alone and calcium plus vitamin D), number of study participants (≤1000 and >1000), daily mean dietary calcium intake (<700 mg/d, 700–1000 mg/d, and >1000 mg/d), and number of low risk of bias (<5 items and ≥5 items). Additionally, in order to clarify the influence of individual trials on the summary effect estimate, a leave-one-out sensitivity meta-analysis was performed. We also compared the differences in the main findings and conclusions between the previous systematic reviews and meta-analyses and the current study.

### 2.5. Statistical Analyses

To estimate a pooled relative risk (RR) with its 95% confidence interval (CI), we calculated a pooled RR with its 95% CI by using values in cells of a 2 × 2 table based on an intention-to-treat analysis, whenever possible. For testing the heterogeneity across the studies, we used Higgins I^2^, which measures the percentage of total variation across publications [13]. I^2^ was computed as follows:

I^2^ = 100% × (Q − df)/Q

where Q is Cochran’s heterogeneity statistic and df indicates the degrees of freedom. Negative values of I^2^ were set at zero; the I^2^ results are between 0% (no observed heterogeneity) and 100% (maximal heterogeneity). An I^2^ value greater than 50% was considered to indicate substantial heterogeneity [13]. A random-effects model meta-analysis on the basis of the DerSimonian and Laird method was used in the current study because individual trials were carried out in the different populations.

We estimated the publication bias using the Begg’s funnel plot and Egger’s test. When publication bias exists, Begg’s funnel plot exhibits asymmetry or the *p*-value < 0.05 by Egger’s test. We used the Stata SE version 13.1 software package (StataCorp, College Station, TX, USA) for the statistical analysis.

## 3. Results

### 3.1. Identification of Relevant Studies

**Figure 1:**
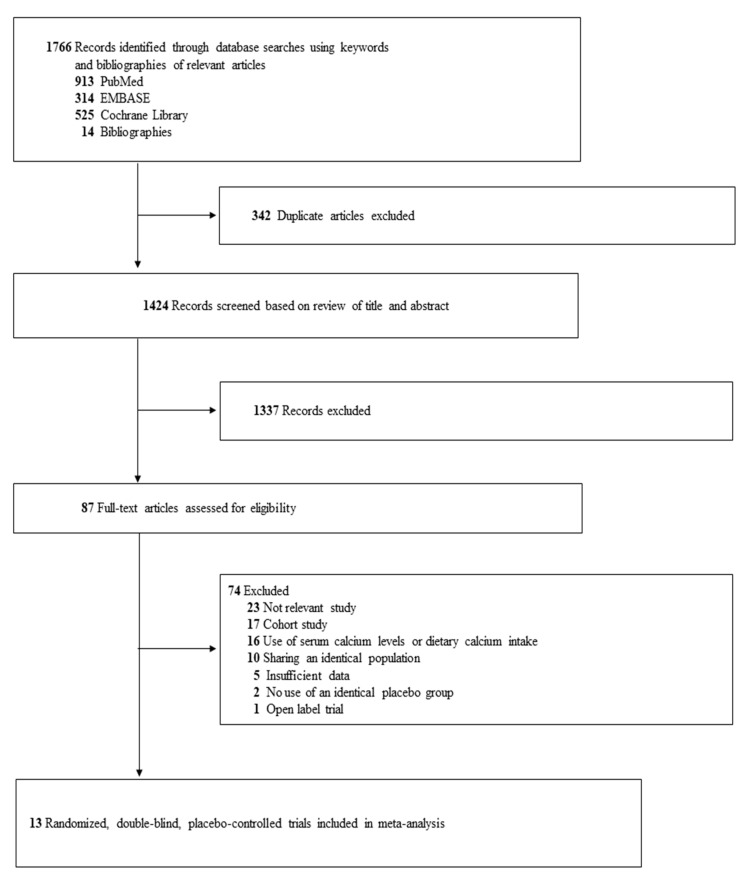
Study selection.

As shown in Figure 1, out of a total of 1495 articles identified by the initial search of three databases and hand-searching relevant bibliographies, 13 double-blind, placebo-controlled RCT [14,15,16,17,18,19,20,21,22,23,24,25,26] were included in the final analysis.

### 3.2. General Characteristics of Trials 

Table 1 shows the general characteristics of 13 double-blind, placebo-controlled RCTs included in the final analysis. The included trials were published between 1990 and 2013, and they involved a total of 28,935 participants (14,692 in an intervention group and 14,243 in a control group). For trials reporting age and sex, the mean age of the study participants was 66.3 years (range, 35 to 97 years), and 92.8% of those were women. Seven trials [16,18,19,22,24,25,26] demonstrated low risk of bias in at least five out of seven items of the Cochrane Risk of Biazs tool, while the remaining six [14,15,17,20,21,23] demonstrated low risk of bias in fewer than five items (Table S1).

### 3.3. Association between the Use of Calcium Supplements and Risk of CVD, CHD, and Cerebrovascular Disease 

**Figure 2:**
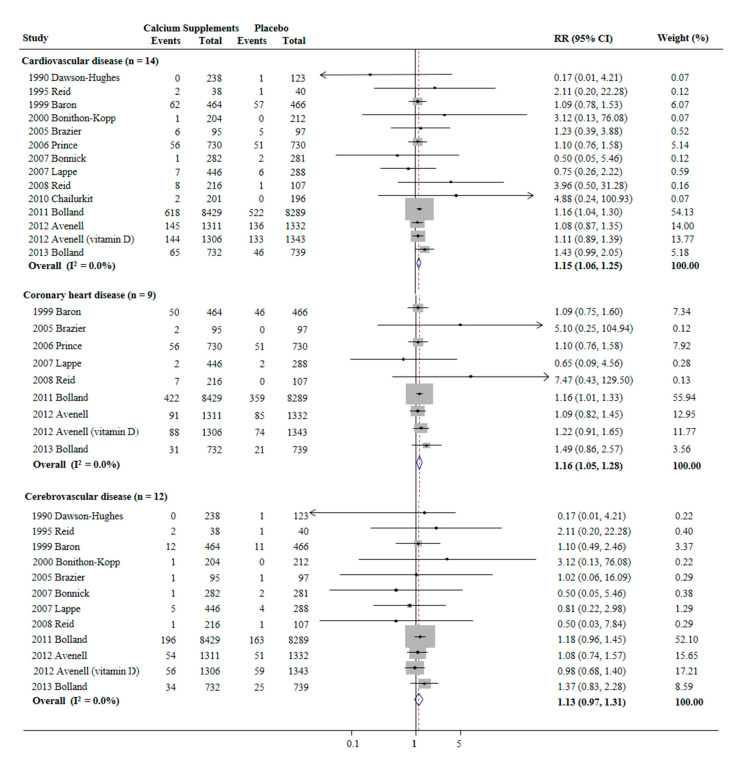
Use of calcium supplements and risk of cardiovascular disease in a random-effects meta-analysis of randomized controlled trials. RR, relative risk; CI, confidence interval. Avenell et al.’s trial [25] used both calcium alone and calcium plus vitamin D in the supplementation groups. Thus, a total of 14 trials were included in the analysis.

Avenell et al.’s trial [25] used both calcium alone and calcium plus vitamin D in the supplementation groups. Thus, a total of 14 trials were included in the analysis. A random-effects meta-analysis of double-blind, placebo-controlled RCTs showed that calcium supplementation significantly increased the risks of CVD (RR 1.15, 95% CI 1.06–1.25], I2 = 0.0%, *n* = 14) and CHD (RR 1.16, 95% CI 1.05–1.28], I2 = 0.0%, *n* = 9) (Figure 2). However, the use of calcium supplements was not significantly association with the risk of cerebrovascular disease (RR 1.13, 95% CI 0.97–1.31, I2 = 0.0%, *n* = 12) (Figure 2).

**Figure 3:**
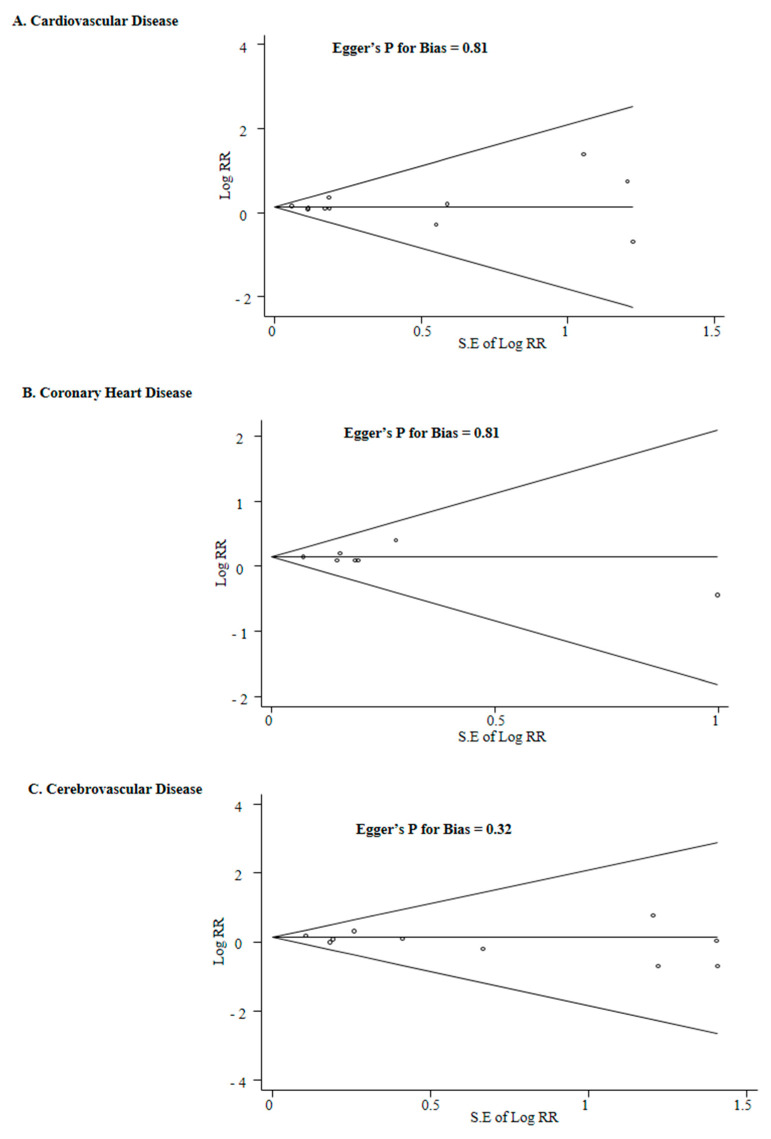
Begg’s funnel plots and Egger’s test for identifying publication bias of randomized controlled trials. RR, relative risk; CI, confidence interval.

Publication bias was not observed: the Begg’s funnel plots were all symmetrical, and *p* for bias was 0.81 for CVD, 0.81 for CHD, and 0.32 for cerebrovascular disease in the Egger’s test (Figure 3).

### 3.4. Subgroup Meta-Analysis

The use of calcium supplements was associated with the increased risk of CVD in the subgroup meta-analysis as follows: incidence, healthy postmenopausal women, women, participants aged <65 years, United States, not specified calcium preparation, duration of calcium supplementation ≥5 years, dosage of calcium supplements ≤1000 mg/d and 1000 mg/d, concurrent use of vitamin D, number of study participants >1000, and daily mean dietary calcium intake of 700–1000 mg/d (Table S2). Remarkably, the trials with low risk of bias in at least five items showed a significantly increased risk of CVD in the calcium supplementation group (RR 1.15, 95% CI 1.06–1.25, I2 = 0.0%, *n* = 8), whereas those with low risk of bias in fewer than five items did not show a significant association with the risk of CVD in the calcium supplementation group.

Similarly, calcium supplementation significantly increased the risk of CHD in the subgroup meta-analysis (Table S3). Like the risk of CVD, the trials with low risk of bias in at least five items showed a significantly increased risk of CHD in the calcium supplementation group (RR 1.16, 95% CI 1.05–1.29, I2 = 0.0%, *n* = 8), whereas the only trial with low risk of bias in fewer than five items did not show a significant association with the risk of CHD in the calcium supplementation group (RR 0.65, 95% CI 0.09–4.56). Additionally, a significant increased risk of CHD in the intervention group was observed in published data (Table S3).

Table S4 shows that, overall, the use of calcium supplements was not associated with the risk of cerebrovascular disease in the subgroup meta-analysis by various factors.

The absolute risk difference for CVD and CHD by using calcium supplements was 8.6 per 1000 persons (0.0086) and 8.8 per 1000 persons (0.0088), respectively.

Figure 3 shows the findings from the leave-one-out sensitivity meta-analysis for each outcome. When Bolland et al.’s study in 2011 was excluded, calcium supplementation was marginally associated with an increased risk of CVD, while all the other leave-one-out sensitivity meta-analyses showed a significantly robust increased risk of CVD (Figure 3A). Additionally, a sensitivity meta-analysis excluding Bolland et al.’s study in 2011 showed that calcium supplementation was not associated with the risk of CHD, while all the other sensitivity meta-analyses did a significantly increased risk of CHD by calcium supplementation (Figure 3B). There was no significant association between calcium supplementation and cerebrovascular disease in any sensitivity meta-analyses (Figure 3C).

### 3.5. Differences among the Previous Systematic Reviews and Meta-Analyses and the Current Study

Table 2 shows the differences in main findings and conclusions among the previous systematic reviews and meta-analyses and the current study. Out of five [4,5,6,10,27] reviews, three [4,5,27] concluded that calcium supplementation increased or might increase the risk of CVD, similar to our findings. On the contrary, in the remaining two reviews, Lewis et al. [6] concluded that calcium supplementation did not increase the risk of CHD, and 2016 Chung et al. [10] concluded that it had a small risk, which was not considered clinically important, if any; moreover, Chung et al. did not perform a meta-analysis on the association between calcium supplementation and the risk of CVD, but rather performed a qualitative review. These inconsistent findings are thought to be attributable to different selection criteria that were used in each meta-analysis, such as the type of study, study population, and inclusion of unpublished data. Only Lewis et al.’s review, which concluded no increased risk of CVD by calcium supplementation, included open-label trials with no use of placebos or no treatment as a control group. Thus, for example, regarding the risk of myocardial infarction, Bolland et al., Bolland et al., Mao et al., and our research reported similar RRs, ranging from 1.24 to 1.28, whereas Lewis et al. obtained an RR of 1.08. Additionally, Chung et al. included only generally healthy adults as study participants and did not include unpublished data. Thus, they included only four trials, which was too small compared to the other reviews. Chung et al. declared that their study was supported by an unrestricted educational grant from the NOF through Pfizer Consumer Healthcare.

## 4. Discussion

We found that calcium supplements were significantly associated with an increased risk of CVD and CHD in a meta-analysis of 28,935 participants from 13 double-blind, placebo-controlled RCTs, specifically in healthy postmenopausal women. In the subgroup meta-analysis, dietary calcium intake of 700–1000 mg per day or supplementary calcium intake of 1000 mg per day significantly increased the risk of CVD and CHD. There was no significant association between the use of calcium supplements and the risk of cerebrovascular disease.

There are several possible biological mechanisms that could explain the increased risk of CVD with the use of calcium supplements observed in our meta-analysis of double-blind, placebo-controlled RCTs. Calcium supplementation could elevate circulating calcium concentrations and, consequently, lead to the development of CVD. First, clinical trials reported that oral calcium supplementation raised serum calcium concentrations [29], which remained elevated after long-term administration of calcium supplements [30]. Furthermore, a randomized controlled trial found that serum calcium levels increased higher after skim milk powder enriched with calcium carbonate supplements than skim powder enriched with milk calcium [31], which might indicate that the intakes of calcium supplements such as calcium carbonate have larger biological effects than dietary calcium intakes such as dairy products. For instance, two meta-analyses of prospective cohort studies [32,33] concluded that dietary calcium intake was not associated with the risk of CVD by comparing the highest and lowest intake levels, while one of them [30] also reported a non-linear association between dietary calcium intake and the risk of CVD mortality. Second, elevated calcium concentrations after calcium supplementation may increase vascular calcification, which is considered as an established factor for CVD. An in vitro study using human vascular smooth muscle cells reported that elevated calcium or phosphate-induced vascular calcification occurred independently and synergistically [34]. Vascular calcification is considered to be a regulated process involving many inhibitors of crystal formation, especially pyrophosphate [35]. Additionally, it was reported that down-regulation of the calcium-sensing receptor due to increased concentrations of calcium results in increased mineralization of the vascular smooth muscle cell cultures, which is another mechanism of vascular calcification by serum calcium [36]. For instance, a retrospective study found that elevated serum levels of calcium, even within the normal range, was significantly associated with the presence of calcified coronary atherosclerotic plaque assessed by cardiac computed tomography angiography [37]. Third, another possible mechanism might be blood coagulation. Free ionized calcium is an essential cofactor for several interactions in the coagulation cascade, and it was reported that whole blood clotting time was prolonged in rats with ionized hypocalcemia induced by rapid intravenous citrate infusion [38]. Conversely, Bristow et al. reported that the administration of calcium supplements increased the coagulation index by about 20% in a randomized placebo-controlled trial of postmenopausal women [39]. Lastly, elevated serum calcium levels are associated with the increased risk of CVD. Reid et al.’s systematic review of observational studies demonstrated that there was a positive association between serum calcium and CVD [40]. Additionally, Larsson et al.’s Mendelian randomization study found that a genetic predisposition to higher serum calcium levels increased the risk of coronary artery disease and myocardial infarction [41].

Our findings are consistent with those from the previous two meta-analyses of RDPCTs by Bolland and colleagues [4,5], while the other two meta-analyses of randomized trials by Lewis et al. [6] and Chung et al. [10] reported no association between calcium supplementation and the risk of CVD unlike our findings. Mao et al.’s meta-analysis also reported that calcium supplementation alone might increase the risk of CVD, although the difference was not statistically significant [28]. As mentioned in the results section, these inconsistent findings are thought to be attributable to different selection criteria, such as type of study, study population, and inclusion of unpublished data.

In the subgroup meta-analysis by mean daily dietary calcium intake, dietary calcium intake of 700–1000 mg/d significantly increased the risk of CVD, while there was no significant association between calcium intakes of lower or higher than 700–1000 mg/d intake and the risk of CVD. However, the number of the included trials for those ranges of calcium intakes was too small to draw a conclusion. Additionally, calcium supplements of 1000 mg/d significantly increased the risk of CVD and CHD. Even though calcium supplements with a concentration lower or higher than 1000 mg/d showed a non-significant association with the risk of CVD and CHD, there was a trend of increased risks of CVD. Further trials are warranted to confirm this finding.

Our study has several limitations. First, most of the double-blind, placebo-controlled RCTs included in our analysis were not designed specifically to investigate the effect of calcium supplements on the risk of CVD as the primary endpoint. In general, findings in the secondary endpoint might be due to chance because the design of the trial is not specifically powered to assess it. However, because conducting a double-blind, placebo-controlled RCT by using only the risk of CVD as the primary endpoint is unethical, further double-blind, placebo-controlled RCTs should consider both the incidence of osteoporosis or fractures and CVD as the primary endpoint, not just as the secondary endpoint. Second, we used unpublished data provided by authors for 5 [14,15,17,20,21] out of 13 double-blind, placebo-controlled RCTs, which were obtained from Bolland et al.’s meta-analysis article [4]. This might be associated with selection bias. However, when we performed a subgroup meta-analysis excluding those five trials, a significant increased risk of CVD and CHD in the calcium supplementation group was still observed. Third, the majority of study participants in the RDBCTs (92.8%) were postmenopausal women with a mean age of 66.3 years. Thus, our findings should be applied to only postmenopausal women. Fourth, the current meta-analysis was not registered at PROSPERO. Last, in the leave-one-out sensitivity meta-analysis for each outcome, when Bolland et al.’s study in 2011 was excluded, calcium supplementation turned out to be marginally associated with an increased risk of CVD and non-significantly associated with an increased risk of CHD. This is attributable to a large sample size of Bolland et al.’s study in 2011, which used the data from the Women’s Health Initiative study involving 16,718 study participants. Thus, further large, double-blind, placebo-controlled RCTs are required to confirm our findings.

In spite of these limitations, our findings have a significant implication. It has been reported that up to 50% of older women take calcium supplements in Western countries [27]. However, a recent meta-analysis [42] of 33 RCTs reported that the use of calcium or vitamin D supplementation was not associated with a lower risk of fractures in older adults, and our study found that the use of calcium supplements might have potential CVD risks. Therefore, in terms of ‘precautionary principle’ as well as evidence-based medicine, supplementary calcium intakes should be discouraged.

## 5. Conclusions

The current meta-analysis of double-blind, placebo-controlled RCT showed that the use of calcium supplements was significantly associated with the increased risk of CVD and CHD by 15%, specifically in postmenopausal women. Our findings should be explicitly confirmed by conducting further RDBCTs with CVD outcome measures as well as the incidence of osteoporosis or fractures as the primary endpoints.

## Figures and Tables

**Table 1:** General characteristics of randomized, double-blind, placebo-controlled trials included in final analysis (n = 13).

Source (Study Name)	Country	Study Participants (Mean Age, y; Women, %)	Mean Dietary Calcium Intake	Duration of Supplementation, Y (Follow-Up Period, Y)	Intervention (vs. Placebo)	Outcomes Used	No. of Major Cardiovascular Events/No. of Participants		Data Source
			(Mg/D)				Supplement Group	Placebo Group	
Dawson-Hughes et al., 1990 [14]	United States	361 healthy postmenopausal women (58.4; 100)	406	2 (2)	Elemental calcium 500 mg/d (as citrate or citrate malate)	Stroke incidence: secondary endpoint	0/238	1/123	Unpublished data *
Reid et al., 1995 * [15]	New Zealand	78 healthy postmenopausal women (58.5; 100)	750	2 (4)	Elemental calcium 1000 mg/d (as lactate-gluconate carbonate)	Stroke incidence: secondary endpoint	2/38	1/40	Unpublished data *
Baron et al., 1999 [16] (CPPS)	United States	930 patients with colorectal adenoma (61; 27.7)	880	4 (4)	Elemental calcium 1200 mg/d (as carbonate)	Hospitalization due to cardiac disease or stroke incidence: secondary endpoint	62/464	57/466	Published data
Bonithon-Kopp et al., 2000 [17] (ECPIS)	10 countries **	640 patients with a history of colorectal adenoma (59.1; 36.4)	980	3 (3)	Elemental calcium 2000 mg/d (as carbonate or gluconate)	Stroke incidence: secondary endpoint	1/204	0/212	Unpublished data *
Brazier et al., 2005 [18]	France	192 women with vitamin D deficiency (74.6; 100)	736	1 (1)	Elemental calcium 1000 mg/d (as carbonate) + vitamin D3 800 IU/d	Cardiovascular events incidence: secondary endpoint	6/95	5/97	Published data
Prince et al., 2006 [19] (CAIFOS)	Australia	1460 elderly women (70; 100)	915	5 (5)	Elemental calcium (as carbonate) 1200 mg/d	IHD incidence: secondary endpoint	56/730	51/730	Published data
Bonnick et al., 2007 [20]	United States	710 postmenopausal women with low bone mineral density (66.2; 100)	1240	2 (2)	Elemental calcium 1000 mg/d (as carbonate) plus alendronate 10 mg/d vs. alendronate 10 mg/d	Stroke incidence: secondary endpoint	1/282	2/281	Unpublished data *
Lappe et al., 2007 * [21]	United States	734 healthy postmenopausal women (66.7; 100)	1070	4 (4)	Elemental calcium 1500 mg/d (as carbonate) or 1400 mg/d (as citrate)	MI and stroke: secondary endpoint	7/446	6/288	Unpublished data *
Reid et al., 2008 [22]	New Zealand	323 healthy men (57; 0)	870	2 (2)	Elemental calcium 600 mg/d or 1200 mg/d	CVD events or TIA: secondary endpoint	8/216	1/107	Published data
Chailurkit et al., 2010 [23]	Thailand	336 physically active healthy postmenopausal women (65.8; 100)	375	2 (2)	Elemental calcium 500 mg/d (as carbonate)	CVD incidence: secondary endpoint	2/201	0/196	Published data
Bolland et al., 2011 [24] (WHI)	United States	16,718 postmenopausal women (62.9; 100) with no personal use of calcium: Reanalysis of the WHI	801	7 (7)	Calcium 1000 mg/d + vitamin D3 400 IU/d	Clinical MI or revascularization + stroke: secondary endpoint	618/8429	522/8289	Published data
Avenell et al., 2012 [25] (RECORD)	United Kingdom	5292 subjects with previous low-trauma fracture (77.2; 85)	820	2–5 (5–8)	Elemental calcium 1000 mg/d (as carbonate) with or without vitamin D3 800 IU/d	CVD or Cerebrovascular disease: primary endpoint	145/1311	136/1332	Published data
Bolland et al., 2013 [26] (ACS)	New Zealand	1471 healthy postmenopausal women (74; 100)	857	5 (5)	Elemental calcium 1000 mg/d (as citrate)	MI or stroke: secondary endpoint	65/732	46/739	Published data

Abbreviations: IHD, ischemic heart disease; MI, myocardial infarction; TIA, transient ischemic attack. CPPS; Calcium Polyp Prevention Study; ECPIS, European Cancer Prevention Organisation Intervention Study; CAIFOS, Calcium Intake Fracture Outcome Study; RECORD, Randomised Evaluation of Calcium Or vitamin D; WHI, Women’s Health Initiative; ACS, Auckland Calcium Study. * Unpublished data were provided by authors, which were obtained from Bolland et al.’s meta-analysis article [4]. ** Belgium, Denmark, France, Germany, Ireland, Israel, Italy, Portugal, Spain, and United Kingdom.

**Table 2:** Differences in the main findings and study characteristics among previous systematic reviews and meta-analyses and the current systematic review and meta-analysis of clinical trials on calcium supplementation and the risk of cardiovascular disease.

		2010, Bolland et al. [4]	2011, Bolland et al. [5]	2013, Mao et al. [28]	2015, Lewis et al. [6]	2016, Chung et al. [10]	Current Meta-Analysis
Conclusion on Calcium Supplementation and Risk of CVD		Increase	Increase	Might Increase	Not Increase	Not Associated, Small Risk and Not Clinically Important, if Any	Increase
Main Findings: RR (95% CI), Number of Included Trials (Reference No.) *, Interpretation in Each Article							
Myocardial Infarction (MI)		- 1.27 (1.01–1.59) - 7 (16, 19, 21, 22, 25, 26) - Increased risk	- 1.24 (1.07–1.45) - 8 (16, 19, 21, 22, 24, 25, 26) - Increased risk	- 1.28 (0.97–1.68) - 8 (16, 19, 21, 22, 24, 25, 26) - Non-significantly increased risk	- 1.08 (0.93–1.25) - 8 (19, 21, 24, 25, 26, 2004 Larsen, 2012 Sambrook) - No increased risk	n.a.	- 1.25 (1.07–1.45) - 9 (16, 18, 19, 21, 22, 24, 25, 26) - Significantly increased risk
Stroke		- 1.12 (0.92–1.36) - 8 (1993 Reid, 16, 19, 20, 21, 25, 26) - No increased risk	- 1.15 (1.00–1.32) - 9 (1993 Reid, 16, 19, 20, 21, 24, 25, 26) - Increased risk	- 1.14 (0.90–1.46) - Not specified - Non-significantly increased risk	n.a.	n.a.	- 1.13 (0.97–1.31) - 12 (14–18,20–22,24–26) - Non-significantly increased risk
Cardiovascular disease (CVD): coronary heart disease (CHD) plus stroke		- 1.12 (0.97–1.30) - 8 (1993 Reid, 16, 19, 21, 22, 25, 26) - No increased risk (composite end point of MI, stroke, and sudden death)	- 1.15 (1.03–1.27) - 10 (1993 Reid, 16, 19, 20, 21, 22, 24, 25, 26) - Increased risk (MI or stroke)	- 1.16 (0.97–1.40) - Not specified - Non-significantly increased risk (major CV events)	- 1.02 (0.96–1.09) - 6 (19, 24, 25, 2004 Larsen, 2012 Sambrook) - No increased risk (CHD)	- No meta-analysis performed - 4 (2011 Lewis, 24, 25, 26) - No statistically significant difference	- 1.15 (1.06–1.25) - 14 (14–26) - Significantly increased risk (CHD plus stroke)
Selection Criteria	Type of Trials	Randomized, double-blind, placebo-controlled trials with >1 year of trial duration	Randomized, double-blind, placebo-controlled trials	Randomized, placebo-controlled trials with at least one year of follow-up	Randomized placebo-controlled trials and open-label trials	Randomized controlled trials	Randomized, double-blind, placebo-controlled trials
	Inclusion of Trials with No Use of Placebos	No	No	No	Yes	No	No
	Study Participants	Participants aged >40 years	Participants aged >40 years	Not described	A mean cohort age >50 years	Generally healthy adults	Adults
	Inclusion of Unpublished Data	Yes	Yes	Yes	Yes	No	Yes
Subgroup meta-analysis		Type of endpoints (MI, stroke, and death)	Type of endpoints (MI, stroke, and MI + stroke)	Type of endpoints (major cardiovascular events, MI, stroke) and sex	Type of endpoints (MI, angina pectoris and acute coronary syndrome, chronic coronary heart disease, and all-cause mortality)	n.a.	Type of endpoints (MI, angina pectoris, coronary revascularization, stroke, coronary heart disease, CVD), low risk of bias, population, age, gender, region, dosage, duration of supplementation, and data source (published or unpublished)
Funding Source		The Health Research Council of New Zealand and the University of Auckland School of Medicine Foundation	The Health Research Council of New Zealand and the University of Auckland School of Medicine Foundation	National “Eleven Five” “Significant new drugs creation” special science and technology major, a major national science and technology projects, etc.	Not described	National Osteoporosis Foundation through Pfizer Consumer Healthcare in U.S.	None

* Ref. [15]—1995 Reid et al. [16]—1999 Baron et al. [18]—2005 Brazier et al. [19]—2006 Prince et al. [20]—2007 Bonnick et al. [21]—2007 Lappe et al. [22]—2008 Reid et al. [24]—2011 Bolland et al. (WHI data) (=2006 Jackson et al.), [25]—2012 Avenell et al. [26]—2013 Bolland et al. (=2006 Reid et al.); Bolland et al.’s meta-analysis included Grant et al.’s trial, which is the first report of the RECORD trial. Ref. [25]—2012 Avenell et al. is the long-term follow-up report for the same trial. In Bolland et al.’s meta-analyses in 2010 and 2011, Grant et al.’s trial (=Ref. [25] 2012 Avenell et al.) was counted as two trials because it reported two findings from the RECORD trial calcium vs. placebo arms and calcium plus vitamin D vs. placebo plus vitamin D arms. 2004 Larsen et al. (open-label trial: a non-placebo control group used), 2012 Sambrook et al. (open-label trial a non-placebo control group used); n.a., not available.

## Data Availability

The authors used published data from the individual studies and declare that the data supporting the findings of this study are available within the article.

## Supplementary Materials

The following are available online at https://www.mdpi.com/2072-6643/13/2/368/s1, Table S1: Summary of risk of bias assessment for randomized, double-blind, placebo-controlled trials, Table S2: Use of calcium supplements and risk of cardiovascular disease (CVD) in the subgroup meta-analysis of randomized, double-blind, placebo-controlled trials, Table S3: Use of calcium supplements and risk of coronary heart disease (CHD) in the subgroup meta-analysis of randomized, double-blind, placebo-controlled trials, Table S4: Use of calcium supplements and risk of cerebrovascular disease in the subgroup meta-analysis of randomized, double-blind, placebo-controlled trials (*n* = 13).
