# Supplementary material for: Calcium Supplements and Risk of Cardiovascular Disease: A Meta-Analysis of Clinical Trials
Source: Nutrients. 2021 Jan 26;13(2):368. doi: 10.3390/nu13020368 (PMC7910980; doi:10.3390/nu13020368)
Supplement: Supplementary file 1 [file nutrients-13-00368-s001.pdf]

Table S1. Summary of Risk of Bias Assessment for Randomized, Double-Blind, Placebo-Controlled Trials.[a]

Source	Random Sequence Generation	Allocation Concealment	Blinding of Participants, and Personnel	Blinding of Outcome Assessment	Incomplete Outcome Data	Selective Reporting	Other Bias	No. of Low Risk of Bias
Dawson-Hughes et al,[14] 1990	Unclear	Unclear	Low	Unclear	Unclear	Unclear	Low	2
Reid et al,[15] 1995	Unclear	Unclear	Low	Unclear	Low	Unclear	Low	3
Baron et al,[16]1999	Low	Unclear	Low	Low	Low	Low	Low	6
Bonithon-Kopp,[17] 2000	Unclear	Low	Low	Unclear	Low	Unclear	Low	4
Brazier et al,[18] 2005	Low	Unclear	Low	Low	Low	Low	Low	6
Prince et al,[19] 2006	Low	Unclear	Low	Low	Unclear	Low	Low	5
Bonnick et al,[20] 2007	Unclear	Unclear	Low	Unclear	Low	Unclear	Low	3
Lappe et al,[21] 2007	Low	Unclear	Unclear	Unclear	High	Low	Low	3
Reid et al,[22] 2008	Low	Low	Low	Low	High	Low	Low	6
Chailurkit et al,[23]2010	Unclear	Unclear	Low	Unclear	Low	Low	Low	4
Bolland et al,[24] 2011	Low	Unclear	Low	Low	Unclear	Low	Low	5

Avenell et al,[25] 2012	Low	Low	Low	Low	Unclear	Low	Low	6
Bolland et al,[26] 2013	Low	Unclear	Low	Low	Low	Low	Low	6

[a]Based on the Cochrane Risk of Bias Tool.

Table S2. Use of Calcium Supplements and Risk of Cardiovascular Disease (CVD) in the Subgroup Meta-analysis of Randomized, Double-Blind, Placebo Controlled Trials.

Factors	No. of Trials	Summary RR (95% CI)	Heterogeneity, I^2 (%)
All	14[14-26]	1.15 (1.06-1.25)	0.0
CVD outcome			
Incidence	12[14-24,26]	1.17 (1.06-1.29)	0.0
Mortality	2[25]	1.10 (0.94-1.28)	0.0
Population			
Healthy postmenopausal women	7[14,15,19,21,23,24]	1.17 (1.06-1.30)	0.0
Subjects with underlying disease (vitamin D deficiency, low bone mineral density, previous low-trauma fracture, or colorectal adenoma)	6[16-18,20,25]	1.10 (0.95-1.26)	0.0
Subjects with underlying disease excluding colorectal adenoma	4[18,20,25]	1.10 (0.94-1.28)	0.0
Subjects with colorectal adenoma	2[16,17]	1.11 (0.79-1.54)	0.0
Gender			
Men	1[22]	3.96 (0.50-31.28)	NA
Women	9[14,15,18-21,23,24,26]	1.17 (1.06-1.30)	0.0
Both	4[16,17,25]	1.10 (0.95-1.27)	0.0

**Factors**	**No. of Trials**	**Summary RR (95% CI)**	**Heterogeneity, I^2 (%)**
Mean age			
<65 years	6[14-17,22,24]	1.16 (1.04-1.29)	0.0
≥65 years	8[18-21,23-26]	1.13 (0.99-1.29)	0.0
Region			
Oceania (New Zealand Australia)	4[15,19,22,26]	1.28 (0.99-1.65)	0.0
North America (United States)	5[14,16,20,21,24]	1.15 (1.03-1.28)	0.0
Europe (Belgium, Denmark, France, Germany, Ireland, Israel, Italy, Portugal, Spain, and United Kingdom)	4[17,18,25]	1.10 (0.94-1.29)	0.0
Asia (Thailand)	1[23]	4.88 (0.24-100.93)	NA
Type of calcium preparation			
Carbonate	6[16,18,19,23,25]	1.10 (0.97-1.26)	0.0
Citrate	2[14,26]	0.92 (0.17-4.93)	39.7
Mixed	3[15,17,21]	1.00 (0.39-2.56)	0.0
Not specified	3[20,22,24]	1.17 (1.04-1.30)	0.0
Duration of calcium supplementation			
<5 years	11[14-23,25]	1.10 (0.96-1.26)	0.0
≥5 years	3[19,24,26]	1.18 (1.06-1.31)	0.0

Factors	No. of Trials	Summary RR (95% CI)	Heterogeneity, I^2 (%)
Dosage of calcium supplements			
≤1000 mg/d	10[14,15,18,20,22,23-26]	1.16 (1.06-1.26)	0.0
500-600 mg/d	3[14,22,23]	1.85 (0.27-12.52)	33.5
1000 mg/d	7[15,18,20,24-26]	1.16 (1.06-1.26)	
>1000 mg/d	5[16,17,19,21,22]	1.11 (0.87-1.40)	0.0
Concurrent use of vitamin D			
Calcium alone	12[14-17,19-23,25,26]	1.13 (1.00-1.28)	0.0
Calcium + Vitamin D	3[18,24,25]	1.15 (1.04-1.27)	0.0
Number of study participants			
≤1000	9[14-18,20-23]	1.11 (0.82-1.49)	0.0
>1000	5[19,24-26]	1.15 (1.06-1.26)	0.0
Mean dietary calcium Intake			
≤700 mg/d	2[14,23]	0.96 (0.04-25.39)	55.5
700-1000 mg/d	10[15-19,22,24-26]	1.15 (1.06-1.25)	0.0
>1000 mg/d	2[20,21]	0.70 (0.26-1.88)	0.0
No. of low risk of bias (out of 7 items)			
<5 items	6[14,15,17,20,21,23]	0.92 (0.41-2.08)	0.0

Factors	No. of Trials	Summary RR (95% CI)	Heterogeneity, I^2 (%)
≥5 items	8[16,18,19,22,24-26]	1.15 (1.06-1.25)	0.0
Data source			
Published data	9[16,18,19,22,23,25,26]	1·15 (1.06-1.25)	0.0
Unpublished data provided by authors	5[14,15,17,20,21]	0.81 (0.35-1.89)	0.0

NA, not applicable; RR, relative risk; CI, confidence interval; CVD, cardiovascular disease; BMI, body mass index.
Avenell et al's trial (Ref.43) used both calcium alone and calcium plus vitamin D in the supplementation groups. Thus, the Avenell et al's trial was separated into two trials (calcium alone vs. placebo and calcium plus vitamin D vs. vitamin D), and the number of trials for Ref.43 is 2.

Table S3. Use of Calcium Supplements and Risk of Coronary Heart Disease (CHD) in the Subgroup Meta-analysis of Randomized, Double-Blind, Placebo Controlled Trials.

Factors	No. of Trials	Summary RR (95% CI)	Heterogeneity, I^2 (%)
All	9[16,18,19,21,22,24-26]	1.16 (1.05-1.28)	0.0
Incidence	8[16,18,19,21,22,24,26]	1.16 (1.03-1.31)	0.0
Mortality	2[25]	1.15 (0.94-1.41)	0.0
Type of CHD			
Myocardial infarction	8[16,18,19,21,22,24,25,26]	1.25 (1.07-1.45)	0.0
Angina	1[22]	1.49 (0.06-36.35)	NA
Coronary revascularization	2[22,24]	1.15 (0.99-1.34)	0.0
Population			
Healthy postmenopausal women	4[19,21,24,26]	1.16 (1.03-1.32)	0.0
Subjects with underlying disease (vitamin D deficiency, previous low-trauma fracture, or colorectal adenoma)	4[16,18,25]	1.14 (0.95-1.37)	0.0
Subjects with underlying disease excluding colorectal adenoma	3[18,25]	1.16 (0.94-1.42)	0.0
Subjects with colorectal adenoma	1[16]	1.09 (0.75-1.60)	0.0
Gender			
Men	1[22]	7.47 (0.43-129.50)	NA

Factors	No. of Trials	Summary RR (95% CI)	Heterogeneity, I^2 (%)
Women	5[18,19,21,24,26]	1.16 (1.03-1.32)	0.0
Both	2[16,25]	1.14 (0.95-1.36)	0.0
Mean age			
<65 years	3[16,22,24]	1.15 (1.01-1.31)	0.0
≥65 years	6[18,19,21,25,26]	1.17 (0.99-1.38)	0.0
Region			
Oceania (Australia and New Zealand)	3[19,22,26]	1.23 (0.91-1.66)	0.0
North America (United States)	3[16,21,24]	1.15 (1.01-1.30)	0.0
Europe (France and United Kingdom)	2[18,25]	1.16 (0.94-1.42)	0.0
Type of calcium preparation			
Carbonate	5[16,18,19,25]	1.13 (0.96-1.33)	0.0
Citrate	1[26]	1.49 (0.86-2.57)	NA
Mixed	1[21]	0.65 (0.09-4.56)	NA
Not specified	2[22,24]	1.77 (0.39-7.18)	39.2
Duration of calcium supplementation			
<5 years	6[16,18,21,22,25]	1.15 (0.96-1.37)	0.0
≥5 years	3[19,24,26]	1.16 (1.03-1.32)	0.0

Factors	No. of Trials	Summary RR (95% CI)	Heterogeneity, I^2 (%)
Dosage of calcium supplements			
≤1000mg/d	6[18,22,24-26]	1.17 (1.05-1.31)	0.0
500-600 mg/d	1[22]	4.95 (0.24-101.99)	n.a.
1000 md/d	5[18,24-26]	1.17 (1.05-1.31)	0.0
>1000mg/d	4[16,19,21,22]	1.10 (0.85-1.42)	0.0
Concurrent use of vitamin D			
Calcium alone	6[16,19,21,22,25,26]	1.13 (0.95-1.36)	0.0
Calcium + Vitamin D	3[18,24,25]	1.14 (1.01-1.29)	0.0
Number of study participants			
≤1000	4[16,18,20,22]	1.15 (0.74-1.80)	2.3
>1000	5[19,24-26]	1.16 (1.04-1.29)	0.0
Mean dietary calcium Intake			
700-1000 mg/d	8[16,18,19,22,24-26]	1.16 (1.05-1.29)	0.0
>1000 mg/d	1[21]	0.65 (0.91-4.56)	0.0
No. of low risk of bias (out of 7 items)			
<5 items	1[21]	0.65 (0.09-4.56)	0.0
≥5 items	8[16,18,19,22,24-26]	1.16 (1.05-1.29)	0.0
Data source			

Factors	No. of Trials	Summary RR (95% CI)	Heterogeneity, I^2 (%)
Published data	8[16,18,19,22,24-26]	1.16 (1.05-1.29)	0.0
Unpublished data provided by authors	1[21]	0.65 (0.09-4.56)	0.0

NA, not applicable; RR; relative risk; CI, confidence interval; CHD, coronary heart diseas.
Avenell et al's trial (Ref.43) used both calcium alone and calcium plus vitamin D in the supplementation groups. Thus, the Avenell et al's trial was separated into two trials (calcium alone vs. placebo and calcium plus vitamin D vs. vitamin D), and the number of trials for Ref.43 is 2.

Table S4. Use of Calcium Supplements and Risk of Cerebrovascular Disease in the Subgroup Meta-analysis of Randomized, Double-Blind, Placebo Controlled Trials (n = 13).

Factors	No. of Trials	Summary RR (95% CI)	Heterogeneity, I^2 (%)
All	12[14-18,20-22,24-26]	1.13 (0.97-1.31)	0.0
Incidence	10[14-18,20,21,22,24,26]	1.18 (0.99-1.42)	0.0
Mortality	2[25]	1.02 (0.79-1.32)	0.0
Population			
Healthy postmenopausal women	5[14,15,21,24,26]	1.19 (0.99-1.44)	0.0
Subjects with underlying disease (vitamin D deficiency, low bone mineral density, previous low-trauma fracture, or colorectal adenoma)	6[16-18,20,25]	1.03 (0.81-1.31)	0.0
Subjects with underlying disease excluding colorectal adenoma	4[18,20,25]	1.01 (0.78-1.31)	0.0
Subjects with colorectal adenoma	2[16,17]	1.17 (0.53-2.55)	0.0
Gender			
Men	1[22]	0.50 (0.03-7.84)	NA
Women	7[14,15,18,20,21,24,26]	1.19 (0.98-1.43)	0.0
Both	4[16,17,25]	1.04 (0.81-1.32)	0.0
Mean age			
<65 years	6[14-17,22,24]	1.17 (0.96-1.43)	0.0
≥65 years	6[18,20,21,25,26]	1.07 (0.85-1.34)	0.0

Factors	No. of Trials	Summary RR (95% CI)	Heterogeneity, I^2 (%)
Region			
Oceania (New Zealand)	3[15,22,26]	1.35 (0.83-2.20)	0.0
North America (United States)	5[14,16,20,21,24]	1.16 (0.95-1.41)	0.0
Europe (Belgium, Denmark, France, Germany, Ireland, Israel, Italy, Portugal, Spain, and United Kingdom)	4[17,18,25]	1.03 (0.80-1.33)	0.0
Type of calcium preparation			
Carbonate	4[16,18,25]	1.03 (0.80-1.32)	0.0
Citrate	2[14,26]	0.91 (0.18-4.61)	36.8
Mixed	3[15,17,21]	1.15 (0.39-3.37)	0.0
Not specified	3[20,22,24]	1.77 (0.95-1.43)	0.0
Duration of calcium supplementation			
<5 years	10[14-18,20,21,22,25]	1.01 (0.80-1.28)	0.0
≥5 years	2[24,25]	1.21 (1.00-1.46)	0.0
Dosage of calcium supplements[a]			
≤1000mg/d	9[14,15,18,20,22,25,26]	1.17 (0.99-1.39)	0.0
500-600 mg/d	2[14,22]	0.17 (0.02-1.62)	0.0
1000 md/d	7[15,18,20,24-26]	1.14 (0.98-1.33)	0.0
>1000mg/d	4[16,17,21,22]	1.05 (0.55-2.03)	0.0

Factors	No. of Trials	Summary RR (95% CI)	Heterogeneity, I^2 (%)
Concurrent use of vitamin D[b]			
Calcium alone	10[14-17,20,21,22,25,26]	1.07 (0.86-1.33)	0.0
Calcium + vitamin D	3[18,24,25]	1.17 (0.98-1.40)	0.0
Number of study participants			
≤1000	8[14-18,20-22]	0.96 (0.53-1.72)	0.0
>1000	4[24-26]	1.14 (0.98-1.33)	0.0
Mean dietary calcium Intake			
≤700 mg/d	1[14]	0.17 (0.01-4.21)	n.a.
700-1000 mg/d	9[15,16-18,22,24-26]	1.14 (0.98-1.32)	0.0
>1000 mg/d	2[20,21]	0.72 (0.23-2.28)	0.0
No. of low risk of bias (out of 7 items)			
<5 items	5[14,15,17,19,21]	0.86 (0.34-2.19)	0.0
≥5 items	7[16,18,22,24-26]	1.13 (0.98-1.32)	0.0
Data source			
Published data	7[14,18,22,24-26]	1.13 (0.98-1.32)	0.0
Unpublished data provided by authors	5[14,15,17,20,21]	0.86 (0.34-2.19)	0.0

NA, not applicable; RR, relative risk; CI, confidence interval; TIA, transient ischemic attack.

2012 Avenell et al's trial (Ref.43) used both calcium alone and calcium plus vitamin D in the supplementation groups. Thus, the Avenell et al's trial was separated into two trials (calcium alone vs. placebo and calcium plus vitamin D vs. vitamin D), and the number of trials for Ref.43 is 2.
